# Immunoproteomic to Analysis the Pathogenicity Factors in Leukopenia Caused by Klebsiella Pneumonia Bacteremia

**DOI:** 10.1371/journal.pone.0110011

**Published:** 2014-10-16

**Authors:** Haiyan Liu, Zhongle Cheng, Wen Song, Wenyong Wu, Zheng Zhou

**Affiliations:** 1 Department of Critical Care, First Affiliated Hospital of Anhui Medical University, Anhui, Hefei, P.R. China; 2 Department of Clinical Laboratory, First Affiliated Hospital of Anhui Medical University, Anhui, Hefei, P.R. China; 3 Department of Radiology, First Affiliated Hospital of Anhui Medical University, Anhui, Hefei, P.R. China; 4 Department of General Surgery, First Affiliated Hospital of Anhui Medical University, Anhui, Hefei, P.R. China; Hokkaido University, Japan

## Abstract

Incidences of leukopenia caused by bacteremia have increased significantly and it is associated with prolonged hospital stay and increased cost. Immunoproteomic is a promising method to identify pathogenicity factors of different diseases. In the present study, we used immunoproteomic to analysis the pathogenicity factors in leukopenia caused by Klebsiella Pneumonia bacteremia. Approximately 40 protein spots localized in the 4 to 7 pI range were detected on two-dimensional electrophoresis gels, and 6 differentially expressed protein spots between 10 and 170 kDa were identified. Pathogenicity factors including S-adenosylmethionine synthetase, pyruvate dehydrogenase, glutathione synthetase, UDP-galactose-4-epimerase, acetate kinase A and elongation factor tu (EF-Tu). In validation of the pathogenicity factor, we used western blotting to show that Klebsiella pneumonia had higher (EF-Tu) expression when they accompanied by leukopenia rather than leukocytosis. Thus, we report 6 pathogenicity factors of leukopenia caused by Klebsiella pneumonia bacteremia, including 5 housekeeping enzymes and EF-Tu. We suggest EF-Tu could be a potential pathogenicity factor for leukopenia caused by Klebsiella pneumonia.

## Introduction

Bacteremia is defined as the presence of viable bacteria in the bloodstream and it can cause mild to life-threatening illnesses through activation of a series of proinflammatory, antiinflammatory and apoptotic cascades that ultimately result in a disruption of physiologic homeostasis. The incidence of bloodstream infections either of community-acquired origin or of hospital-acquired origin has dramatically increased all over the world [1], [2]. Bacteremia is typically associated with prolonged hospital stay and increased medical cost, particularly if accompanied with leukopenia, since patients with leucopenia have a worse prognosis than infected but non-leukopenia patients [3]–[5]. For example, patients hospitalized as a consequence of community-acquired, health care associated or hospital-acquired pneumonia have increased mortality rates if they were also leukopenia [6].

Researches in bacteremia have identified C-reactive protein,IL-6 and procalcitonin as markers of bloodstream infections [7], [8]. In previous study, we proved that bacteremia induced by pandrug-resistant Klebsiella pneumonia caused less damage compared with bacteremia induced by drug-susceptible Klebsiella pneumonia, but little work have been carried out to find the pathogenicity factors of bacteria which could result into leukopenia [9].

Immunoproteomics, a technique involving two-dimensional electrophoresis followed by immunoblotting, holds considerable promise for the discovery of pathogenicity factors in different diseases, including cancer, autoimmune diseases, and infections [10]–[12]. In this study, we applied an immunoproteomic approach to survey the leukopenia caused by pathogenicity factors of Klebsiella pneumonia and identified 6 pathogenicity factors.

## Method

### Ethical statement

Study protocols were approved by the medical ethics committee of First Affiliated Hospital of Anhui Medical University. Written informed consent was obtained from all subjects prior to participation.

### Bacteria samples and patients serum

Case group consist of Klebsiella pneumonia isolates from patients with Klebsiella pneumonia bacteremia and leukopenia and control group consist of Klebsiella pneumonia isolates from patients with Klebsiella pneumonia bacteremia and leukocytosis. All Klebsiella pneumonia isolates in this study were drug-susceptible isolates. Serum specimens were collected from the patients when they got Klebsiella pneumonia. Bacteria samples and serum samples were kept frozen at −80°C, and thawed just before analysis. All patients were recruited between Jan 2011 and Oct 2013 in the first affiliated hospital of Anhui medicine university, China. Acute Physiology and Chronic Health Evaluation (APACHE) II was calculated according to the initial pathography of patients when they were hospitalized. Leukocyte count and CRP were recorded when the patients got Klebsiella pneumonia. Leukopenia was defined as WBC count <4000/µl. Leukocytosis was defined as WBC count >10000/µl. Patients with underlying diseases (diabetes mellitus, heart disease, et al) and chemotherapy were excluded from the study.

### Preparation of Klebsiella pneumonia lysates

#### Cell Culture

Bacteria were inoculated in liquid Luria-Bertani culture medium and grown 24 h at 37°C. Bacteria density was measured spectrophoto metrically at 600 nm. Bacteria were pelletted by centrifugation (10 min, 20 000 g, 4°C) and washed thrice in sterile PBS (Na2-HPO4 0.01 M; NaCl 0.15 M; 0.05% Tween 20 pH 7.4).

#### Bacterial Cell Lysis

After cell culture, bacterial pellets were lysed for 1 h in the lysis buffer (saccharose 0.78 M, Tris-HCl 30 mM pH 8, EDTA 5 mM, and lysozyme 100 ug/mL). The protein concentration in supernatants was determined by the Bradford method.

### Protein separation by 2-D PAGE

Bacterial lysates of each group was pooled and then Klebsiella pneumonia proteins (60 mg) were diluted with rehydra-tion solution (8 M urea, 2% CHAPS, 65 mM DTT, 0.5% vol/vol isoelectric focusing buffer [pH 4–7], trace bromophenol blue) to a final volume of 250 ml. IEF was performed using a Protean IEF system (Amersham Bioscience, Sweden). Gels were rehydrated at 30 V for 6 hours and at 60 V for 6 hours. Proteins were subsequently focused for 1 hour at 500 V and 1 hour at 1000 V; then, a gradient was applied from 1000 to 8000 V for 1 hour; finally, the voltage was set at 8000 V to subject the samples to a total of 30,000 V h. All IEF experiments were performed at 20 uC.

After one-dimensional IEF, IPG strips were placed in an equilibration solution (6 M urea, 2% sodium dodecyl sulfate [SDS], 30% glycerol, 1.5 M Tris-HCl, pH 8.8) containing 2% DTT and were shaken for 15 minutes at 50 rpm. The strips were transferred to an equilibration solution containing 2.5% iodoace-tamide, shaken for 15 minutes, transferred to vertical slabs of 12.5% SDS-PAGE gels, and sealed with 0.5% low-melting-point agarose. After electrophoresis, proteins in gels were transferred to a PVDF membrane and the rest of proteins in gels were visualized by silver staining. Images were digitized using a GS-800 Calibrated Densitometer (Bio-Rad, USA) and analyzed with the PDQuest 2-D Analysis software package.

### Detection of bacteria pathogenicity factors by Western blotting

After protein transfer, the PVDF membranes were incubated for 2 hours in blocking buffer comprising 5% milk in 10 mM Tris-HCl (pH 7.5), 2.5 mM EDTA (pH 8.0), and 50 mM NaCl. Serum samples from patients were mixed together as the source of primary antibodies at 1∶200 dilution under room temperature (RT) for 2 hours. After 3 washes with washing buffer (Tris-buffered saline containing 0.01% Tween 20), membranes were incubated with goat antihuman IgG antibodies (Beijing Zhongshan Company, China) at a dilution of 1∶5,000 for 1 hour at RT. Pathogenicity factors in cases were compared directly with pathogenicity factors in controls.

### In-gel enzyme digestion and mass spectrometry

Each differentially expressed spot from the gels was dehydrated with 50 ml of ACN for 5 min, incubated in 50 mL of 10 mM DTT at 56 uC for 1 hour, and then incubated in 50 mL of 55 mM iodoacetamide (alkylating solution) for 45 min. The spots were dehydrated with 50 ml of ACN and rehydrated in 5 ml of porcine trypsin, followed by the addition of 10 ml of 25 mM ammonium bicarbonate. Proteolysis was performed overnight at 37 uC and stopped by adding 10 ml of 2% formic acid. Resulting peptides were concentrated and mixed with alpha-cyano-4-hydroxycinam-mic acid (Sigma, St. Louis, MO, USA), deposited on a 384-well MALDI target, and air-dried. Analyses were performed with a Biflex IV (Bruker Daltonics, Germany). MS data were compared against tryptic peptide sequences from the SWISS-PROT database using Mascot (Matrix Sciences, London, UK) search algorithms.

### Western blot verification

New bacteria sample were subjected to 12.5% SDS-PAGE and then transferred onto polyvinylidene difluoride (Millipore, Billerica, MA) membranes, which were blocked for 30 minutes with 5% nonfat milk in Tris-buffered saline with 0.05% Tween-20 (TBST). Samples were incubated overnight at 4°Cin 1∶400 v/v solutions of EF-Tu Ab (Abcam UK). The membranes were washed 3 times in TBST for 5 minutes each and blotted for 2 hours with a 1∶500 solution of HP-conjugated secondary rabbit antimouse IgG at RT. The membranes were washed twice with TBST, followed by another wash in TBS. Immunoreactive bands were visualized with a Western Blotting Detection System (Pierce, Rockford, IL).

## Results

### Patients characteristics

During the study period, 8 patients (6 males and 2 female; median age, 63 years) in case group and 8 patients (5 males and 3 female; median age, 61 years) in control groups were enrolled. There was no difference between the two groups in APACHE II score. In the case group, the mean leukocyte counts was 3.2×10^9^/Land the mean CRP was 37.3 mg/L. In the control group, the mean leukocyte counts was 12.3×10^9^/L and the mean CRP was 87.6 mg/L.

### Klebsiella pneumonia pathogenicity factors

Total protein extracts of Klebsiella pneumonia were separated by 2-D PAGE and transferred to PVDF membranes. Serum samples from patients were mixed. The serum proteins were the primary antibody and goat antihuman IgG was the secondary antibody in our assay. By compared the results in cases and controls, approximately 40 protein spots localized in the 4–7 pI range were detected on the 2-DE gels. Six differentially expressed protein spots between 10 and 170 kDa were identified.

### Identification of the reactive proteins

**Figure 1:**
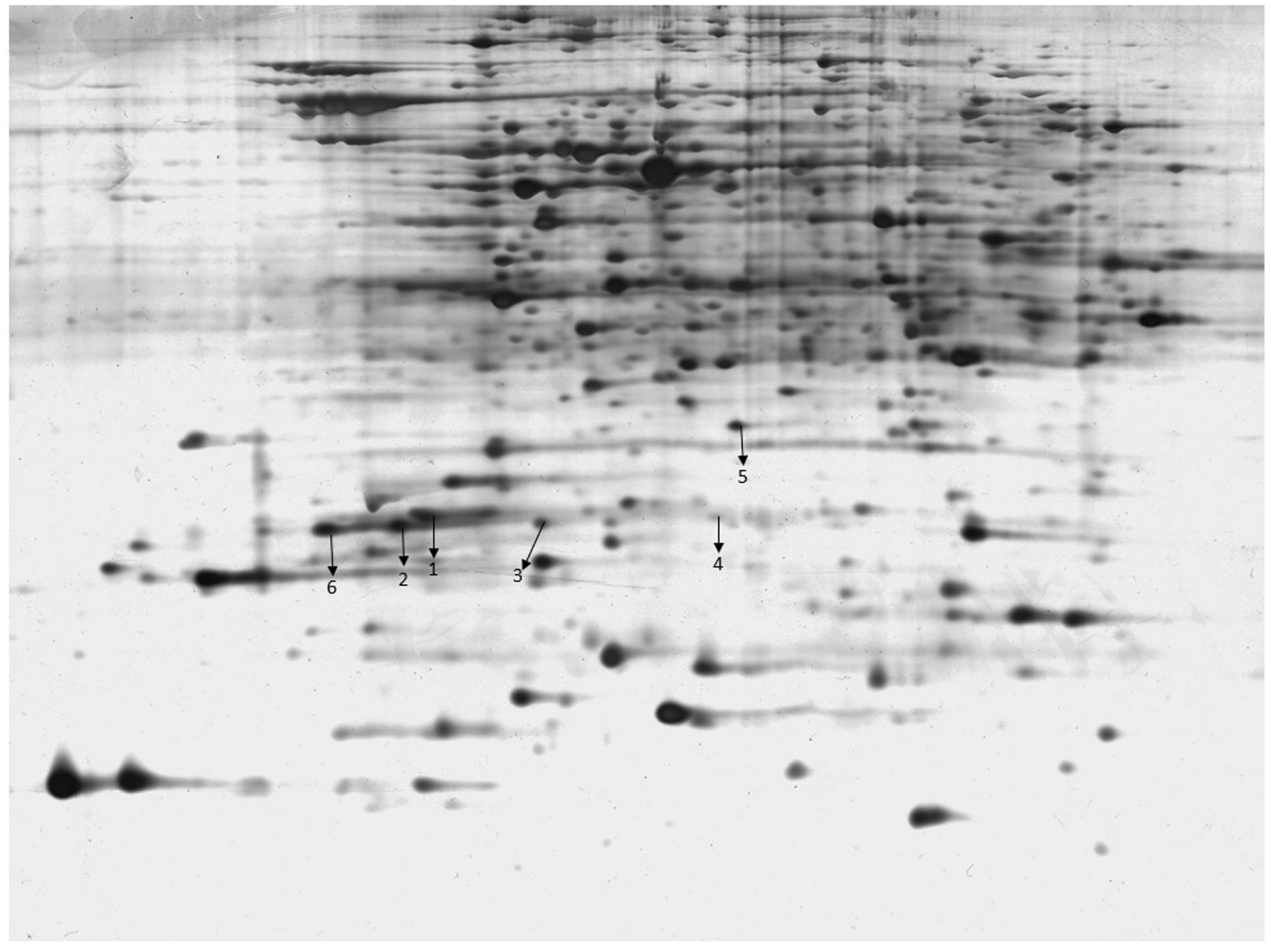
2-DE analysis of proteins from Klebsiella pneumonia in leukocyte patients caused by Klebsiella pneumonia bacteremia. Detection of pathogenicity factors by Western blot analysis in CD patients. The six protein spots are labeled with arrows.

Proteins of interest were extracted from the gels after 2-D PAGE and silver staining. The proteins were digested with trypsin, chymotrypsin, and Glu-C, and the resulting peptides were analyzed by MALDI-TOF MS. The corresponding spectra were used to identify the proteins with the Mascot search program. The reactive proteins were identified as S-adenosylmethionine synthetase, pyruvate dehydrogenase, glutathione synthetase, UDP-galactose-4-epimerase, acetate kinase A and elongation factor Tu (Fig. 1, Table 1).

**Table 1:** Differentially Expressed Proteins Identified by MALDI-TOF-MS.

accession number	Sequence coverage	Score	pI/Mr	Protein name
WP_004133543	22%	92	5.14/39378	S-adenosylmethionine synthetase
WP_004178692	35%	106	5.07/36538	pyruvate dehydrogenase subunit beta
WP_004205176	44%	84	5.19/35656	glutathione synthetase
WP_009308200	58%	192	5.63/37155	UDP-galactose-4-epimerase
WP_004201756	37%	109	5.89/43527	acetate kinase A
WP_004169020	47%	109	4.86/37221	elongation factor Tu, partial

### Western blot analysis to validate the reactive proteins

**Figure 2:**
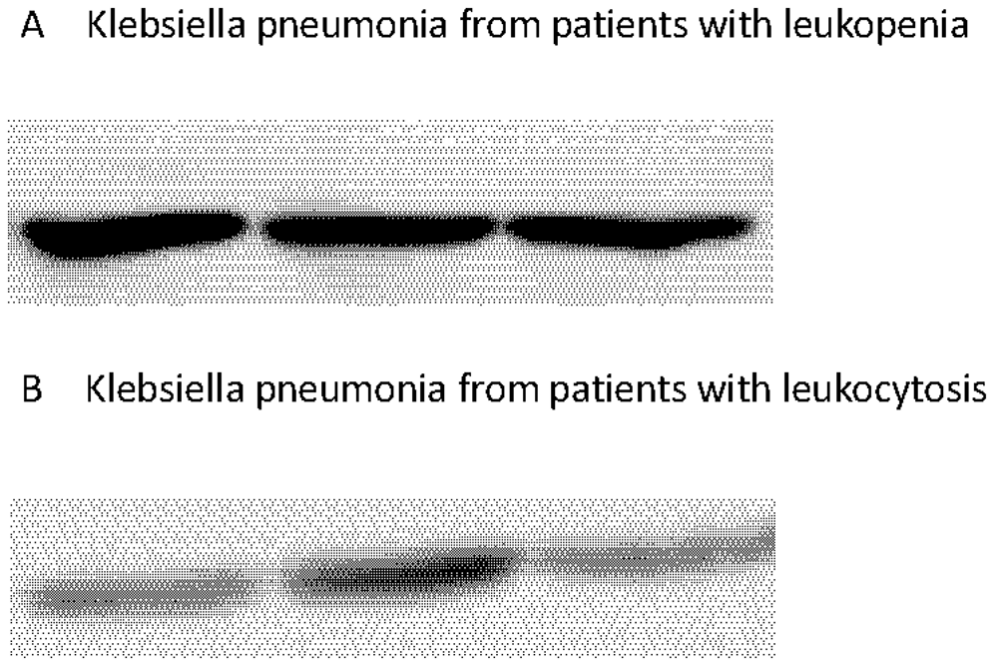
Western blot analysis to validate the reactive proteins. Klebsiella pneumonia from Klebsiella pneumonia bacteremia patients with (**A**) leukopenia and (**B**) leukocytosis were respectively examined by Western blot analysis to validate the reactive proteins ef-tu. Note the higher expression levels of ef-tu in patients with leukopenia caused by Klebsiella pneumonia.

To confirm the immunoproteomic data, another 3 Klebsiella pneumonia isolates from patients with bacteremia and leukopenia were compared with another 3 Klebsiella pneumonia isolates from patients with leukocytosis by Western blot. Drug-susceptible isolates with bacteremia and leukopenia had high levels of elongation factor tu expression (Fig 2A, Fig 2B).

## Discussion

Identification of disease-specific pathogenicity factors is essential for understanding the diseases and defining biomarkers for detection of preclinical disorders. With prolonged hospital stay and increased medical cost from leukopenia induced by bacteremia, it is very important to find out that how bacteremia has caused leukopenia. In this study we revealed 6 pathogenicity factors for leukopenia caused by Klebsiella pneumonia bacteremia. S-adenosylmethionine synthetase, pyruvate dehydrogenase, glutathione synthetase, UDP-galactose-4-epimerase, acetate kinase A are housekeeping enzymes. EF-Tu (elongation factor thermo unstable) is one of the prokaryotic elongation factors. It is part of the mechanism that synthesizes new proteins by translation at the ribosome. Intrinsic differences between Klebsiella pneumonia with respect to differences in protein expression profiles was negated by pooling the lysates of Klebsiella pneumonia isolates from patients, further strengthening the association of these six candidate pathogenicity factors with leukopenia. In addition, the presence of the pathogenicity factor EF-Tu in lysates was confirmed by western blot analysis.Our immunoproteomics approach for proteins profile of Klebsiella pneumonia bacteremia with leukopenia is a convenient and effective tool for detecting pathogenicity factors.

S-adenosyl-L-methionine (SAM) acts as a methyl donor in amino acid, protein, phospholipid, and DNA synthesis. The majority of cellular SAM is used in methylation of macromolecules of prokaryotes and eukaryotes, yielding S-adenosylhomocysteine (SAH) as a product. Quorum Sensing systems regulate pathogen–host cell interactions, bacterial virulence and the formation of bacterial biofilms. SAM is the substrate for the LuxI-type enzymes whch consist of quorum sensing circuits in gram-negative bacterial [13]. Knockout mutants of luxS genes in V. cholerae, S. pyogenes, S. pneumoniae, N. meningitidis and C. perfringens exhibited severe defects in the expression of genes encoding virulence factors, suggestting that inhibitors of MTA/SAH nucleosidase could possibly become new antiinfective drugs against various bacterial pathogens [14].

Pyruvate dehydrogenase is the first component enzyme of pyruvate dehydrogenase complex. The pyruvate dehydrogenase complex contributes to transforming pyruvate into acetyl-CoA by a process called pyruvate decarboxylation.

In a study focused on the activation of the Pseudomonas aeruginosa type III secretion system, which is a recently identified virulence determinant of P.aeruginosa, the results suggest that in the pyruvate dehydrogenase mutants exsA expression is not induced, which does not allow the type III secretion system to function efficiently [15], [16]. In a recent research studying the modes of action of antimicrobial compounds, using an exometabolome profiling approach, Birkenstock identified a unique TPBC(triphenylbismuthdichloride)-mediated change in the metabolites of staphylococcus aureus was, indicating that TPBC blocks bacterial pyruvate catabolism [17].

Glutathione synthetase is the second enzyme in the glutathione biosynthesis pathway. It catalyses the condensation of gamma-glutamylcysteine and glycine, to form glutathione [18]. The role of glutathione synthetase in bacteria is very important, it can protect bacteria from damage of osmotic stress, low pH, toxicity of ethylglyoxal and oxidative stress [19]–[22].UDP-galactose-4-epimerase is an essential enzyme of the Leloir pathway of galactose metabolism and catalyzes the interconversion of UDP-galactose and UDP-glucose [23]. UDP-galactose thus formed serves as galactose donor for the biosynthesis of galactosyl residues in glycoproteins and complex polysaccharides, including both the core and O-antigen polysaccharide of the LPS [24]. Acetate kinase, a conserved enzyme that is widespread in bacteria, is responsible for the phosphorylation of acetate [25]. In a study showed inhibition of Escherichia coli growth by the two inhibitors of acetate kinase, suggests the potential role of acetate kinase could be a potential bacteriostatics target [26].

EF-Tu is a component of the prokaryotic mRNA translation apparatus that delivers aminoacyl-tRNAs to the ribosome during the elongation cycle of protein synthesis [27]. In proteomic profiling of immunodominant spore antigens of Bacillus anthracis, Bacillus cereus, and Bacillus thuringiensis, EF-Tu was identified one of differentially expressed and immunogenic spore proteins [28]. In other study of Burkholderia infection, Nieves et al found active immunization with EF-Tu induced antigen-specific antibody and cell-mediated immune responses in mice. Mucosal immunization with EF-Tu also reduced lung bacterial loads in mice challenged with aerosolized B. thailandensis, showing the utility of EF-Tu as a novel vaccine immunogen against bacterial infection [29]. In a research of Lactobacillus johnsonii, Granato et al found EF-Tu was expressed intracellularly, as well as on the bacterial cell surface where it participated in gut homeostasis through its binding to the intestinal mucosa [30].

However, the limitations of the present study should be acknowledged. First, the number of Klebsiella pneumonia patients was too small to generalize current findings to a large population. As a result, all outcomes need to be confirmed in further study with large sample size. Second, the lack of controlled Klebsiella pneumonia bacteremia with normal leukocyte counts follow up data makes it difficult to choose more accurate pathogenicity factors. In conclusion, our proteomic analysis identified 6 housekeeping enzymes and EF-Tu was linked to leukopenia induced by Klebsiella pneumonia bacteremia. We suggest EF-Tu could be a potential pathogenicity factor for leukopenia caused by Klebsiella pneumonia.
